# MicroRNA-552 enhances metastatic capacity of colorectal cancer cells by targeting a disintegrin and metalloprotease 28

**DOI:** 10.18632/oncotarget.12169

**Published:** 2016-09-21

**Authors:** Jian Wang, Hai Li, Yong Wang, Libin Wang, Xiurui Yan, Dong Zhang, Xiaoqiang Ma, Yong Du, Xiaoming Liu, Yinxue Yang

**Affiliations:** ^1^ The general hospital, Ningxia Medical University, Yinchuan 750004, China; ^2^ Human Stem Cell Institute of the General Hospital at Ningxia Medical University, Yinchuan 750004, China; ^3^ Department of Colorectal Surgery, the General Hospital of Ningxia Medical University, Yinchuan 750004, China

**Keywords:** microRNAs, colorectal cancer, miR-552, antagomir, ADAM28

## Abstract

Colorectal cancer (CRC) is one of the most common prevalent cancer types worldwide. MicroRNAs (miRNAs or miRs) have been demonstrated to play crucial roles in the development, metastasis and drug resistance of CRC. In the present study, a strikingly elevated expression of miR-552 was determined in CRC tumor tissues and cells by a miRNA profiling analysis. Importantly, the gene of A Disintegrin And Metalloprotease (ADAM) family member 28 (ADAM28) was identified as a target of miR-552, which was further validated in terms of genetic dual luciferase report assay. Furthermore, an inhibition of miR-552 in LOVE and LS174T CRC cells by transducing miR-552 inhibitor (antagomiR-552) with a lentiviral vector exhibited an ability to reduce cell proliferation, migration and clonogenicity. Moreover, both LOVO and LS174T cells stably expressing miR-552 inhibitor displayed a decreased ability to develop tumors in a murine xenograft model *in vivo*. In contrast, a knockdown of ADAM28 by short hairpin RNA could reverse the antagomiR-552-induced inhibition of metastatic features of CRC cells *in vitro*. These results suggested that miR-552 is an oncomir able to promote CRC metastasis in part through a mechanism of targeting ADAM28, which may be a novel target for CRC treatment and warrants for further investigation.

## INTRODUCTION

MicroRNAs (miRNAs) are a family of 18–22 nt small non-coding RNAs that negatively regulate the gene expressions of target mRNAs at the post-transcriptional level through a non-genetically mutational mechanism. To date, accumulating evidence has demonstrated the importance of miRNAs in cancer development and progression. In this regard, the dysregulated miRNAs have been involved in the regulation of gene function that contributed to the initiation, growth, metastasis, recurrence and acquisition of drug-resistance in a variety of cancers, including the colorectal cancer (CRC). CRC is the third most common malignant neoplasm worldwide, which accounts for 9.7% of all cancer incidences [1–5]. Indeed, recent miRNA profiling studies have demonstrated alterations of miRNA expression in tumors compared to paired adjacent normal tissues in CRC, which has been proposed to correlate with the stages and metastasis in CRC patients. In this context, miRNAs can play a functionality of either tumor-suppressors or oncogenes (oncomirs) through mechanisms of directly targeting genes in the key steps of initial and metastatic processes, as well as acquired drug-resistances [6–8].

A disintegrin and metalloproteinases (ADAMs) are a new gene family of proteins that structurally classified into two groups, the membrane-anchored ADAM [9] and ADAM with thrombospondin motifs (ADAMTS) [10]. To date, 34 and 21 of ADAM family members are respectively identified in mouse and human, these members are characterized with sequence similarity to the reprolysin family of snake venomases that share the metalloproteinase domain with matrix metalloproteinases (MMPs) [11]. A compelling body of studies have indicated that ADAM members have diverse roles in various biological processes, such as the cell adhesion, cell fusion, cell migration, cell proliferation and the pathogenesis of a variety of diseases, particularly in cancers [12–14]. In this regard, the expression of many ADAM species, including ADAM8, ADAM9, ADAM10, ADAM17, ADAM28, ADAMTS4 and ADAMTS5 are up-regulated in several types of human tumors involved in the regulation of cell proliferation and malignancy, despite the precise mechanism of their oncogenic roles remains incompletely understood [13]. For example, ADAMTSs secreted by cancer and stromal cells may contribute to modify tumor microenvironments, where ADAMTSs are implicated in the cell invasion, migration, proliferation and angiogenesis *via* mechanisms involved in cleaving or interacting with extracellular matrix components or regulatory factors [12]. Among these ADAMs, ADAM8, 9, 10, 12, 15, 17, 19, 22, 23 and 28 have been demonstrated to play a regulatory role in the initiation, procession and metastasis of cancers [13].

A disintegrin-metalloproteinase 28 (ADAM28) is one of important members of ADAM family, which consists of two isoforms, prototype membrane-type form (ADAM28m, 775 amino acids) and short secreted form (ADAM28s, 540 amino acids), and has been involved in various biological events including cell adhesion proteolysis, growth and metastasis of solid tumors and hematological malignancies [15]. Accumulated lines of evidence have shown that ADAM28 expression was strikingly up-regulated in several human cancers [16], such as non-small cell lung cancer [17–19], breast cancer [20], bladder cancer [21] and chronic lymphocytic leukemia [22]. In addition, its expression in cancer cells was correlated with the metastasis of cancers [16]. For instance, ADAM28 was the most frequent and selective ADAM species expressing in the breast and lung carcinoma tissues, and the abundance of its transcripts was directly correlated with the capacity of cell proliferation and metastasis [19, 20]. Mechanistically, the oncogenic role of ADAM28-mediated cancer cell metastasis may be related with its ability to cleave factors including von Willebrand's factor (vWF) [15], insulin-like growth factor binding protein-3 (IGFBP-3) [23], and connective tissue growth factor (CTGF) [24], and to promote PSGL-1/P-selectin-mediated cell adhesion [25].

In the CRC, the correlation of ADAM28 and CRC tumorigenesis has not yet been established, although transcripts of ADAM28 and IGFBP-3 genes in fresh CRC tumor specimens were primary examined in CRC patients with overweight or obese using a microarray analysis [23]. In consistent with findings in other cancer types, the change of ADAM28 and IGFBP-3 genes expression was only observed in normal tissues but not tumor tissues of overweight/obese patients with CRC, implying that alterations of the expression of ADAM28 and IGFBP-3 may be an initial process of cancer proliferation, despite the histopathologically normal surgical margin in this group of patients was not equal to the molecular margin [23]. In normal tissues, ADAM28 may play a protective role in cell survival. For instance, a recent study demonstrated that the ADAM28 played a role in cell survival of bronchial epithelial cells by suppressing a C1q-induced cytotoxicity [26].

Several lines of evidence have demonstrated that ADAMs could be regulated by miRNAs in various cancers [27–29], and we and others have recently revealed a strikingly up-regulated miR-552 and miR-592 in CRC tissues as compared to the matched adjacent non-tumor tissues, which imply the it may play a oncogenic role in CRC tumorigenesis [30, 31] and metastasis [32, 33]. In this regard, miR-552 was found to correlate with the clinical stage, lymph node and distant metastases, as well as chemoresistance of CRC [34]. By using the online computational miRNA target prediction tool, TargetScan (http://www.targetscan.org), ADAM28 was predicted as a potential target of miR-552. Together with the fact of that no miRNA has been reported to target ADAM28 yet, we therefore hypothesize that the ADAM28 might ba a target of miR-552 in CRC.

## RESULTS

### Evoked miR-552 and miR-592 transcripts in human colorectal cancer

**Figure 1:**
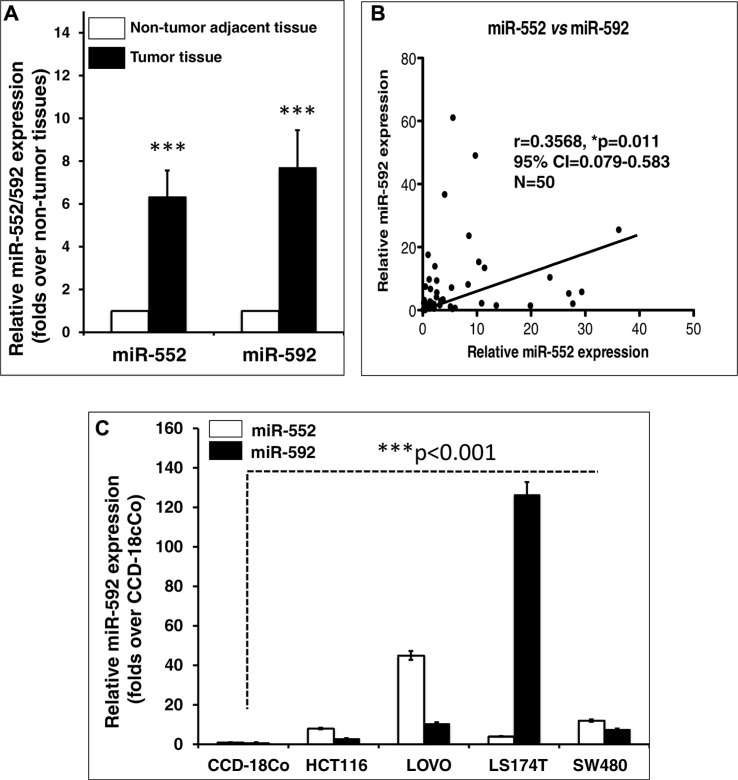
Aberrant miR-552 and miR-592 transcripts in colorectal cancer tissues and cell lines (**A**) Relative expression of miR-552 and miR-592 in CRC tissues. Both miR-552 and miR-592 expression is significantly elevated in CRC tissues as compared with the matched adjacent non-tumor tissues (*p* < 0.001). (**B**) Correlation of miR-552 and miR-592 in CRC tissues (*r* = 0.3568, 95% CI = 0.079–0.583, *N* = 50). (**C**) Relative expression of miR-552 and miR-592 in CRC cell lines. Both microRNAs were up-regulated in CRC cell lines HCT116, LOVO, LS174T and SW480, as compared with the CCD-18Co normal intestinal epithelial cell line (*p* < 0.001). Data expressed the mean ± SD; ***p < 0.001.

Previous miRNA microarray analysis has demonstrated that miR-552 and miR-592 were an oncomir and up-regulated of in CRC [30, 31, 33, 35, 36]. In order to further validate a correlation of the expression of these miRNAs and clinicopathologic stages in CRC, the relative expression of miR-552 and miR-592 in CRC tumor tissues and cell lines was evaluated by a qRT-PCR assay (Figure 1 and Table 1). In line with the previous reports from other groups, results of this study also displayed a significantly more abundant miR-552 and miR-592 transcripts in tumor tissues relative to the matched adjacent non-tumor tissues (Figure 1A and Table 1), and the expression of miR-552 was also correlated with the abundance of miR-592 transcript in CRC tissues (*r* = 0.3568, 95% CI = 0.079–0.583, *p <* 0.011, *N* = 50) (Figure 1B). In addition, all examined CRC cell lines, including HCT116, LOVE, LS174T and SW480, also showed an elevated expression of miR-552 and miR-592 in comparison with the normal colon epithelial cell line CCD-18Co (Figure 1C). Particularly, LOVO and LS174T cells showed the most and least abundance of miR-552 transcript among the examined cell lines, respectively. Therefore they were chosen as cell models for further investigations in this study. Since the oncogenic role of miR-592 in CRC has been extensively investigated [33, 35, 36], the biological significance of miR-552 in CRC was mainly focused in this study. Interestingly, an up-regulated miR-552 was also correlated with the TNM stages and lymph node metastasis in CRC (Table 1).

**Table 1:** Relative miR-552 and miR-592 expression in tumor tissues compared to the matched adjacent non-tumor tissues of various stages of colorectal cancer

Subjects	Ages	Gender	Degrees of differentiation	TNM stages	Lymph node metastasis	Relative miR-552 expression$	Relative miR-592 expression$
CRC01	64	M	Low	ΙI	No	1.432	6.71
CRC02	74	M	Low	ΙI	No	1.808	2.01
CRC03	56	F	Low	ΙII	Yes	0.481	1.39
CRC04	63	M	Low	ΙII	Yes	5.479	0.45
CRC05	75	F	Low	ΙV	Yes	0.089	0.08
CRC06	68	M	Low	ΙΙ	No	1.24	1.87
CRC07	47	F	Low	ΙΙΙ	Yes	3.395	3.04
CRC08	64	M	High	ΙΙ	No	19.88	1.40
CRC09	75	M	High	Ι	No	0.484	7.47
CRC10	62	F	High	ΙΙΙ	Yes	26.95	5.30
CRC11	57	M	High	ΙΙΙ	Yes	8.376	8.14
CRC12	77	F	High	ΙΙ	No	9.723	49.06
CRC13	64	F	High	IV	Yes	23.475	10.39
CRC14	75	M	High	III	Yes	10.337	15.31
CRC15	66	M	High	II	Yes	11.409	13.43
CRC16	57	M	Medium	II	No	1.193	9.78
CRC17	62	M	Medium	I	No	1.535	0.78
CRC18	32	M	Medium	III	Yes	1.367	2.75
CRC19	57	M	High	II	No	4.06	36.72
CRC20	64	M	High	II	No	1.308	0.54
CRC21	76	M	High	II	No	5.31	7.14
CRC22	50	M	Medium	II	No	5.131	1.13
CRC23	55	M	High	II	No	1.349	1.98
CRC24	59	F	Medium	II	No	2.077	1.29
CRC25	61	F	Medium	III	Yes	1.672	2.33
CRC26	74	M	Medium	II	No	0.283	3.03
CRC27	77	M	Medium	II	No	0.529	0.14
CRC28	59	M	High	III	Yes	10.883	2.19
CRC29	47	F	High	IV	Yes	0.181	2.33
CRC30	61	M	Low	III	Yes	0.994	17.59
CRC31	48	M	High	II	No	27.71	2.04
CRC32	49	M	Medium	III	Yes	13.574	1.46
CRC33	63	F	Medium	III	Yes	5.578	61.08
CRC34	76	M	Medium	II	No	29.351	5.82
CRC35	82	F	Medium	IV	No	0.261	3.17
CRC36	47	M	Medium	I	No	2.538	4.14
CRC37	62	M	Medium	I	No	2.095	0.52
CRC38	64	M	Medium	III	Yes	0.383	0.07
CRC39	82	F	Medium	II	No	1.121	1.92
CRC40	64	F	Medium	II	No	3.342	2.88
CRC41	62	F	Medium	II	No	2.095	1.89
CRC42	59	F	Medium	III	Yes	1.715	1.18
CRC43	46	F	Medium	II	No	8.523	23.6
CRC44	59	M	Medium	II	No	3.73	3.35
CRC45	81	M	High	II	Yes	2.586	9.39
CRC46	76	M	Low	II	Yes	2.259	13.9
CRC47	32	M	Medium	III	Yes	36.183	25.5
CRC48	57	M	Medium	I	No	3.161	1.6
CRC49	77	M	Low	II	No	5.946	0.71
CRC50	77	M	Low	II	No	2.592	5.63

$ Fold of miR-552 and miR-592 transcript in tumor tissues over the corresponding adjacent non-tumor tissue. M: male; F: female; MA: mucoid adenocarcinoma Data of miRNA qRT-PCR was performed with the 2^−ΔΔ CT^ method against CRC adjacent non-tumor tissues.

### The expression of ADAM28 is suppressed in human colorectal cancer

**Figure 2:**
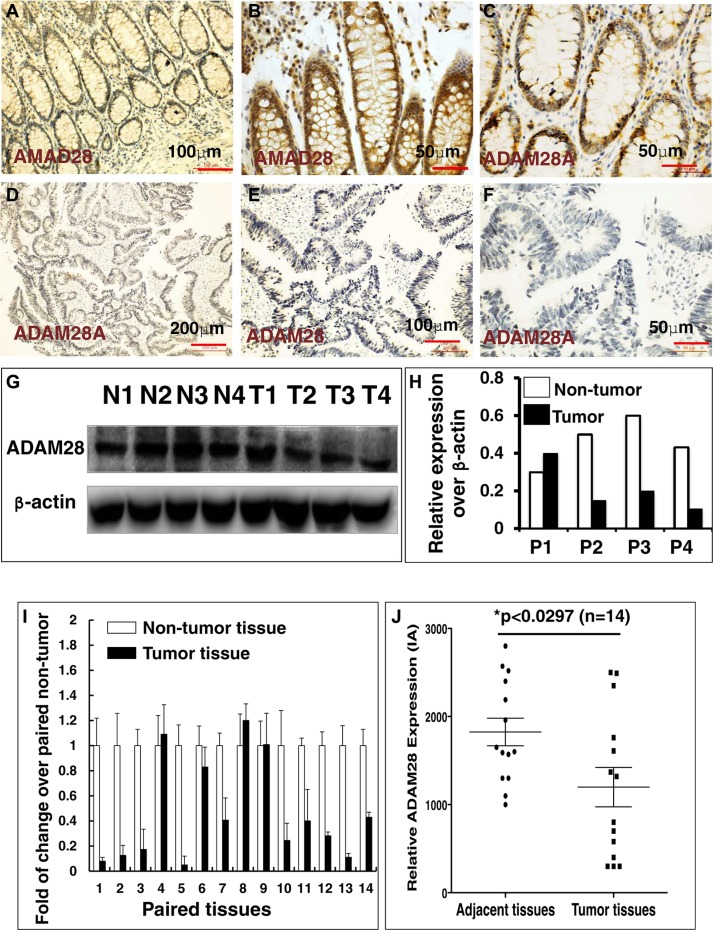
Immunohistochemistry (IHC) staining determined ADAM28 expression in human CRC tumors and matched adjacent tissues (**A**–**F**) Representative images of the expression of ADAM28 protein determined by an IHC staining. (A–C) Images represented the ADAM28 expression in non-CRC tumor adjacent tissues at different magnifications; (D–F) Images represented the ADAM28 expression in CRC tumor tissues at different magnifications. (**G**) Immunoblotting assay determined ADAM28 protein in 4 paired CRC tissues and non-tumor intestinal tissues (T = CRC tumor tissue; N = non-tumor intestinal tissues). (**H**) Semi-quantitative analysis of ADAM28 protein expression in the 4 paired tumor and non-tumor tissues in the Figure 4G by densitometry assay. Data represented ratio over respective loading control b-actin (P: Patient with CRC). (**I**) Relative expression of ADAM28 mRNA in paired CRC tumor tissues and the match non-tumor adjacent tumor tissues (*N* = 14). (**J**) Semi-quantitative analysis of ADAM28 protein expression using integrated absorbance (IA) in human CRC tissues and the matched adjacent non-tumor tissues. Value was expressed as the average values from each individual sample of CRC tumor tissues or its matched adjacent tissue. The total average value of IA in the CRC tumor tissues was significantly less abundant as compared with the matched adjacent tissues (*p* < 0.05, *n* = 14). Data was expressed as mean ± SD for 14 sets of samples. Bar in A, E: 100 μm; in B, C, F: 50 μm; D: 200 μm.

In order to interrogate the biological significances of miR-552 in the tumorigenesis of CRC, the online computational miRNA target prediction tool, TargetScan (http://www.targetscan.org), was used for identifying potential targets of miR-552 (Supplementary Table S1). The ADAM28 was screened as a potential target of miR-552 for further investigation, partially owing to a conserved seed sequence of this miRNA was possessed within the 3′UTR of its mRNA, and its crucial role in pathogenesis of many types of cancers, including the CRC, in which ADAM28 expression was correlated with a poor prognosis [22, 23, 37, 38], despite its role in CRC remained unclear [23]. To further explore the clinical relevance of ADAM28 with the clinic pathogenesis in human CRC, its expression was first evaluated in CRC tumor tissues and the matched adjacent non-tumor tissues by IHC staining against anti-ADAM28 antibody. The result exhibited a more pronounced ADAM28 protein in non-tumor tissues (Figure 2A–2C), as compared to the matched tumor tissues (Figure 2D–2F). This finding was further supported by the immunoblotting assay, by which much less abundant ADAM28 protein was detected in CRC tumor tissues relative to the matched non-tumor tissues (Figure 2G and 2H). Notably, the strikingly suppressed expression of ADAM28 in CRC tumors was confirmed by a semi-quantitative assay using a RT-PCR assay (Figure 2I) and a measurement of the integrated absorbance (IA) of IHC images from twenty examined paired archival CRC samples (*p <* 0.05) (Figure 2J and data not shown), suggesting that ADAM28 may play a tumor suppressor role in the carcinogenesis of CRC, and is an attractive potential target for treatment of this disease [16].

### Validation of ADAM28 mRNA as a target of miR-552

**Figure 3:**
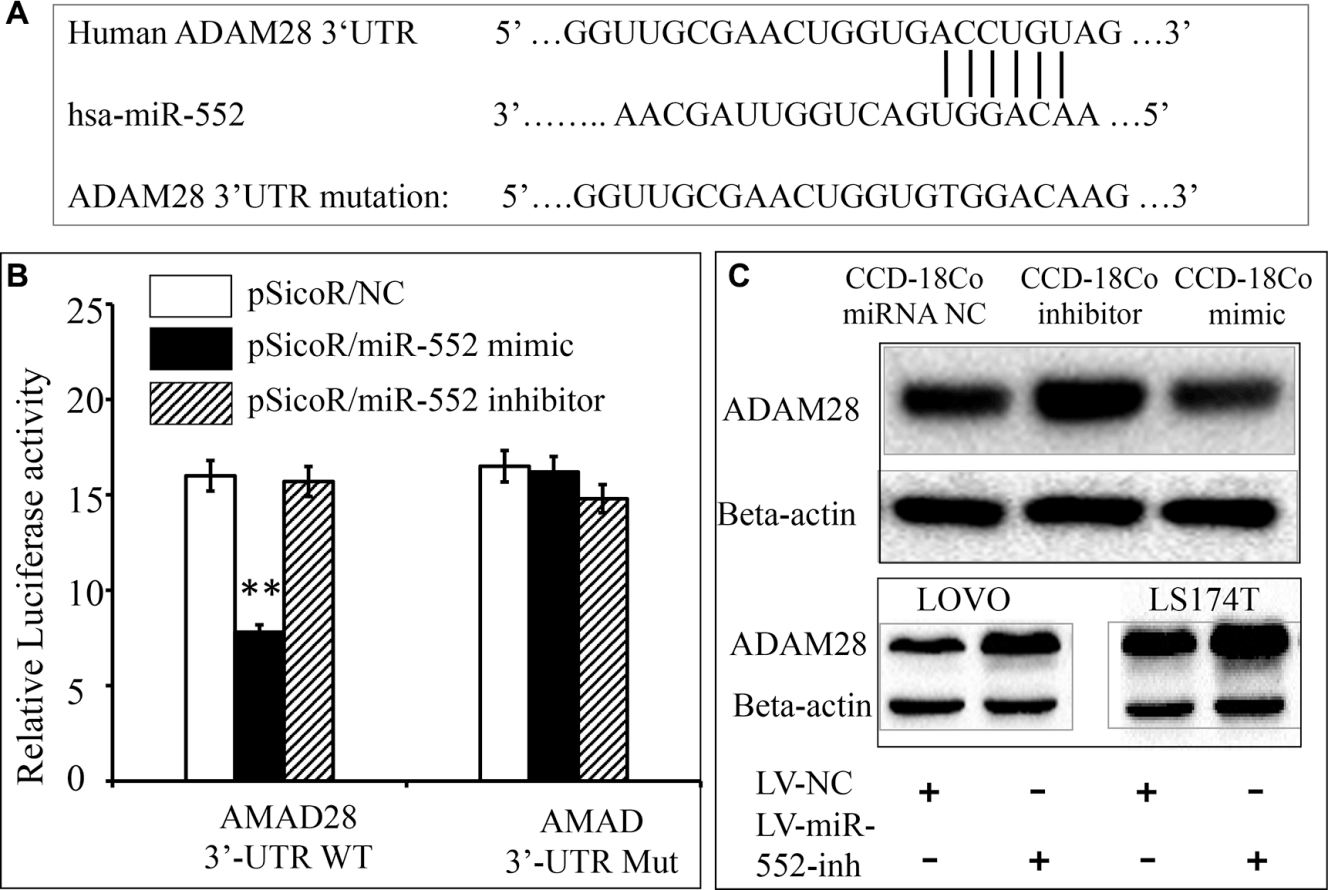
Validation of ADAM28 mRNA as a target of miR-552 (**A**) Sequence of potential binding site of miR-552 in the 3′UTR of human ADAM28 mRNA (top sequence), mutations were introduced into the binding site for generation of mutated ADAM28 3′TUR (bottom sequence). (**B**) Validation of miR-552 target using ADAM28 3′UTR luciferase reporter. C: The expression of ADAM28 was detected by immunoblotting in CCD-18Co normal colon epithelial cells transfected with indicated miR-552 control (NC), inhibitor and mimic. The immunoblotting assay showed an increased and decreased abundance of ADAM28 protein in the CCD-18Co cells transfected with the miR-552 inhibitor and mimic, respectively (top panel); in addition, an increased abundance of ADAM28 protein in the both LOVO and LS174T cells infected with the LV-miR-552 inh viral vector as compared to the LV-NC (top panel). **Compared with pSicoR/NC group, *p* < 0.01. Results represented the mean ± SD from three independent triplicated experiments (*N* = 9).

To experimentally validate whether ADAM28 is a potential target of miR-552 in CRC. Luciferase reporter vector containing a 3′UTR of ADAM28 mRNA (pMIR-Report/ADAM28 3′UTR), or a mutated 3′UTR (pMIR-Report/Mut-ADAM28 3′UTR) were first constructed (Figure 3A). The 293T cells were co-transfected with pMIR-Report/ADAM28 3′UTR or pMIR-Report/Mut-ADAM28 3′UTR, and scramble miRNA control (NC), miR-552 mimics, or miR-552 inhibitor. The results of dual luciferase assay showed a significant decrease of relative luciferase activity in the cells transfected with miR-552 mimics, in comparison with the control miRNA or inhibitor transfected cells (Figure 3B). Immunoblotting assay further confirmed that ADAM28 protein in the CCD-18Co cells was increased and decreased when they were transfected with the miR-552 inhibitor and mimic, respectively; in addition, an increased abundance of ADAM28 protein was found in both LOVO and LS174T cells infected with the LV-miR-552-inh viral vector as compared to the LV-NC (Figure 3C). This data further confirmed that ADAM28 might be a potential target for the oncomir miR-552 in CRC.

### miR-552 enhances the capacity of CRC cell migration and proliferation

**Figure 4:**
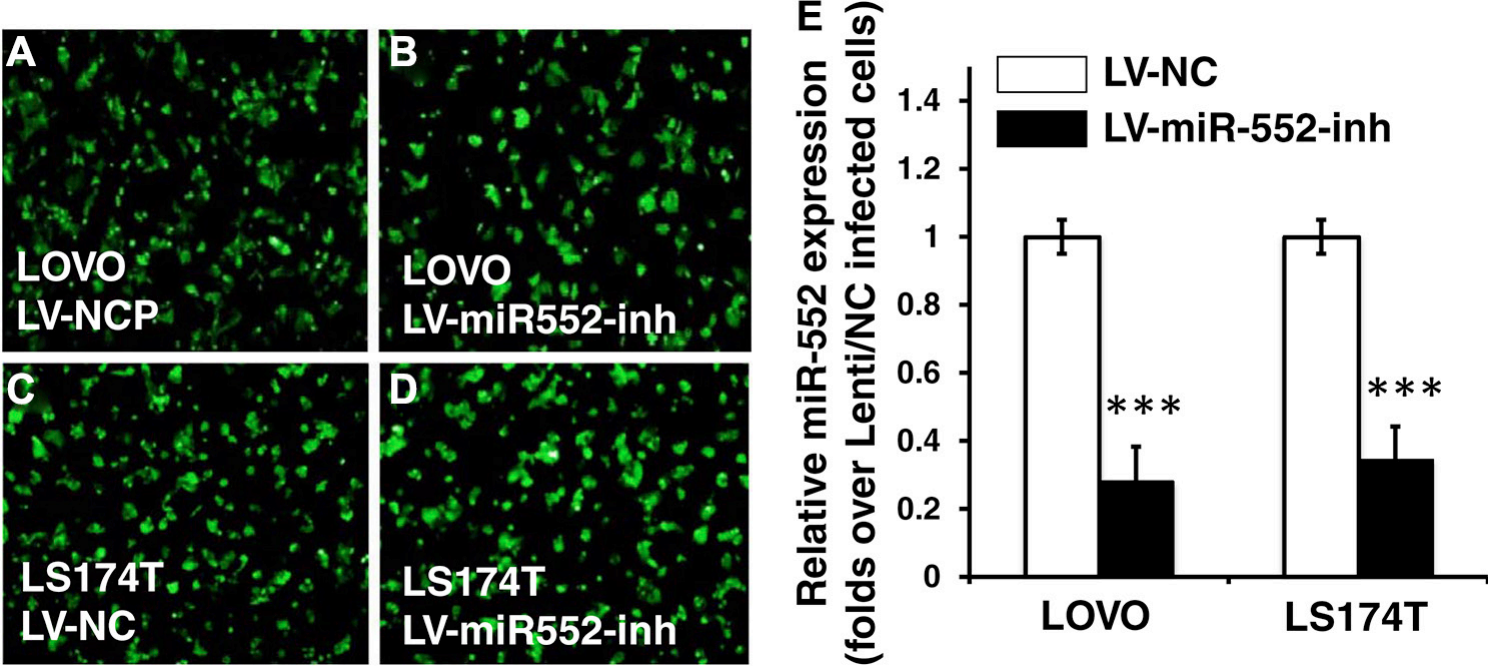
Generation of CRC cell lines expressing miR-552 inhibitor Following a DNA cloning strategy, the proviral plasmids were used for production of lentiviral vector LV-miR-552-inh and LV-NC, which express miR-552 inhibitor and scramble control, respectively. These vectors also expressed an EGFP reporter gene for accessing the transduction efficiency. (**A**–**B**) The LOVO cells transduced with LV-NC (A) and mR-552-inh (B) showed an infectivity of lentiviral vectors. (**C**–**D**) The LS174T cells transduced with LV-NC (C) and mR-552-inh (D) showed an infectivity of lentiviral vectors. (**E**) qRT-PCR result exhibited a reduced abundance of miR-552 transcript in the LV-miR-552-inh-infected cells relative to the LV-NC-infected cells. Data represents the mean ± SD from three independent experiments. Compared to the LV-NC-infected group, ***p < 0.001.

**Figure 5:**
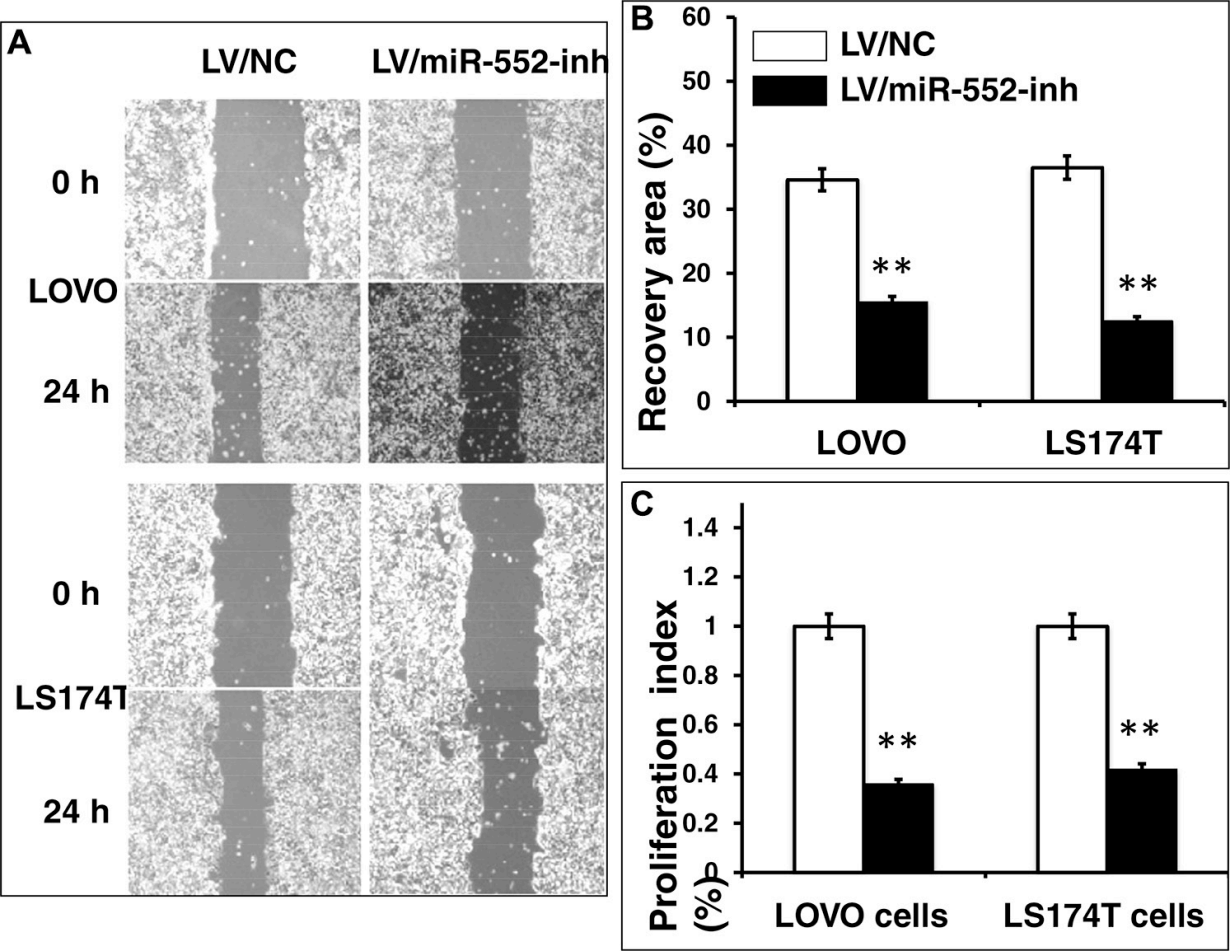
Inhibition of miR-552 reduces LOVO and LS174T CRC cell migration and proliferation *in vitro* LOVO and LS174T cells were infected with LV-miR-552-inh or LV-NC, the capability of cell migration was accessed in terms of a scratch assay, and cell proliferative ability was ascertained by an MTT method. (**A**) Representative images of scratch assays for LOVO cells (top two panels) and LS174T cells (bottom two panels) treated as indicated condition. (**B**) Relevant quantification of the results of cell migration index. (**C**) An MTT assay showed an inhibition of cell proliferation in LV-miR-552-inh-infected cells. Compared with LV-NC group, **p < 0.01. Data in B and C represented the mean ± SD from three independent triplicated experiments (*N* = 9).

Since miR-552 was up-regulated in CRC cells, in order to explore its biological role in the development and metastasis of CRC, lentiviral vectors expressing miR-552 inhibitor and a control scramble miRNA, lentiviral vectors LV-miR-552-inh (antagomiR-552) and LV-NC were produced, respectively. These viruses were used to infect LOVO and LS174T CRC cells, or generate cell lines stably expressing antagomiR-552 or a scramble miRNA control (Figure 4A–4D). A significantly reduced miR-552 transcript was observed in cells infected with LV-miR-552-inh, as compared with those transduced with LV-NC vectors as determined by a qRT-PCR assay (Figure 4E). Intriguingly, the migration capacity in both miR-552-inh-transduced LOVO and LS174T cells was significantly reduced in comparison with those infected with LV-NC virus, as determined using a scratch assay (*p <* 0.01) (Figure 5A–5B). Consistently, the proliferation of CRC cells was also dramatically inhibited when the cells were enforced expression of the antagomiR-552, relative to the cell transduced with NC scramble miRNA, as determined by an MTT method (*p <* 0.01) (Figure 5C).

### miR-552 enhances clonogenicity and tumorigenicity of CRC cells

**Figure 6:**
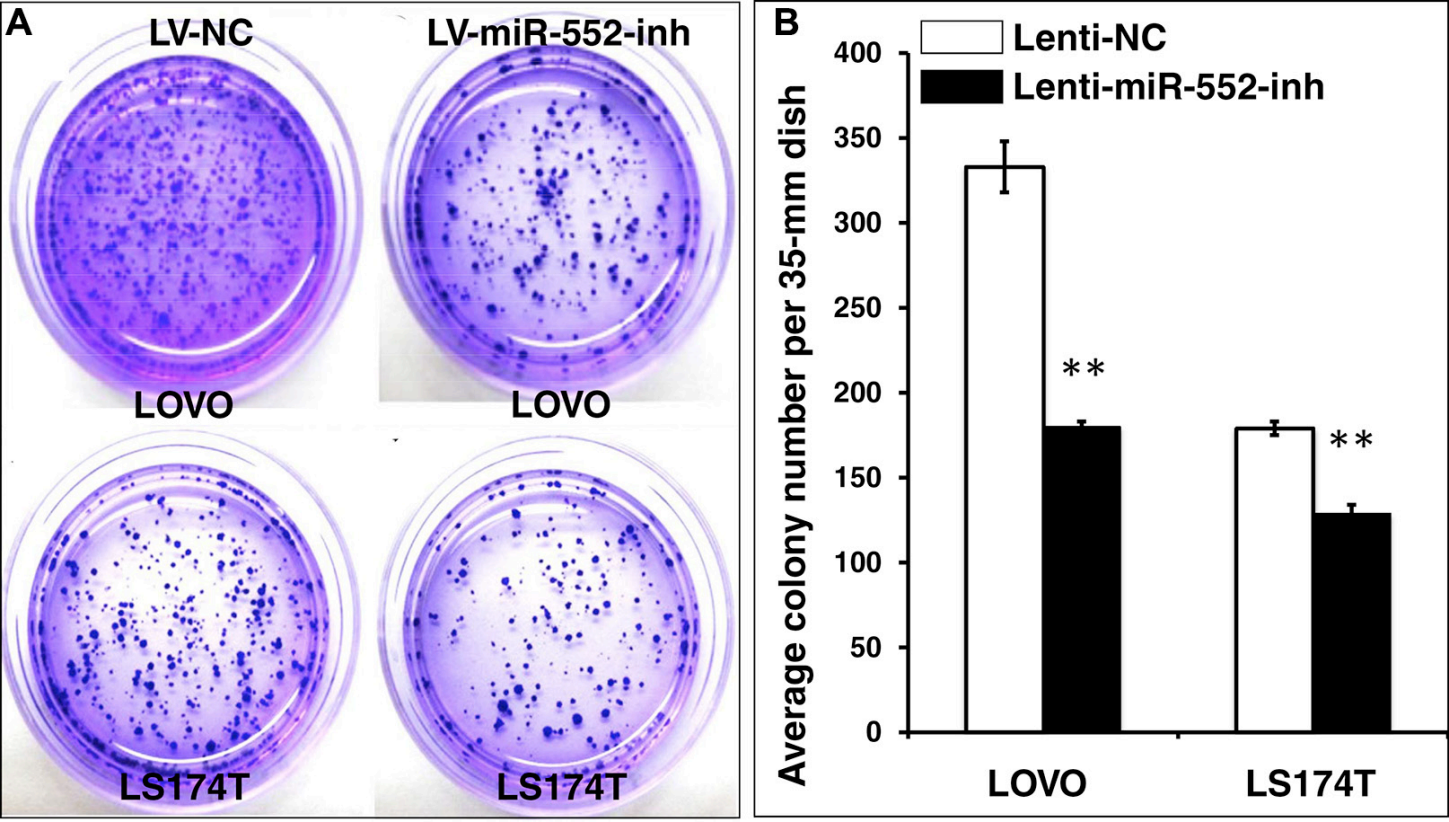
Inhibition of miR-552 suppresses the clonogenicity in LOVO and LS174T CRC cells LOVO and LS174T cells stably expressing miR-552-inh or miRNA NC were the formation of cell colonies using a clonogenic assay in 35-mm dishes. (**A**) Representative images of clonogenic assay for LOVO cells (top panel) and LS174T cells (bottom panel). (**B**) Relevant quantification of the results of cell clonogenic index, which showed an inhibition of clonogenic capacity in LV-miR-552-inh-infected cells. Compared with LV-NC group, **p < 0.01. Data in B represented the mean ± SD from three independent triplicated experiments (*N* = 9).

**Figure 7:**
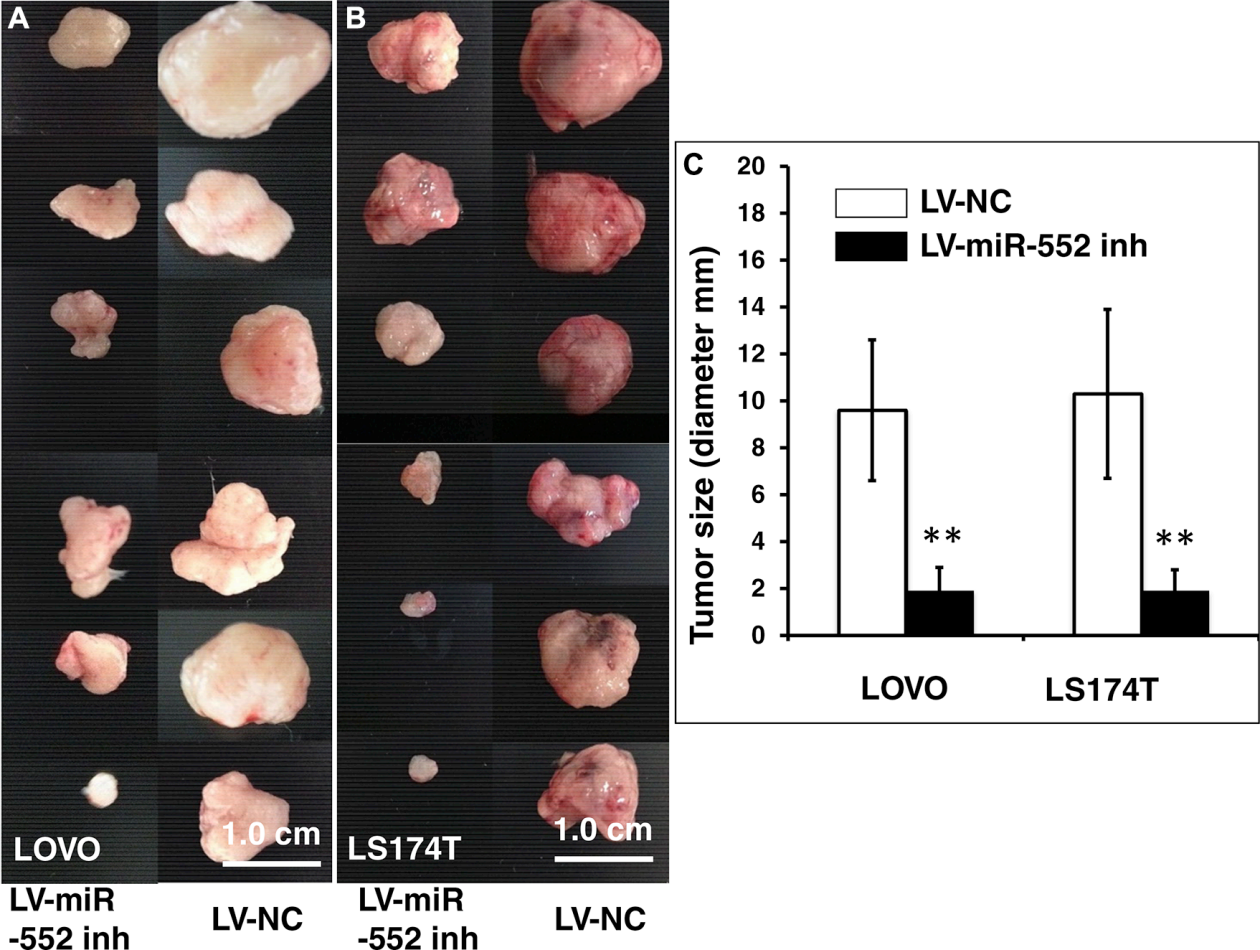
*In vivo* tumorigenic analysis of LV-miR-552-inh-infected CRC cells SCID mice were subcutaneously injected 200 μl Matrigel containing 10^6^ LV-infected LOVO or LS174T cells, and the formation of tumor was at three weeks after the injection. (**A**) Images showed sizes of tumors formed from LOVO cells infected with LV-miR-552-inh (top panel) and LV-NC (bottom panel). (**B**) Images showed sizes of tumors formed from LS174T cells infected with LV-miR-552-inh (top panel) and LV-NC (bottom panel). (**C**) Relevant quantification of the sizes (diameter) of tumors derived from CRC cells expressing miR-552-inh. The result showed an ability of miR-552 inhibitor to reduced tumor sizes. Compared with LV-NC group, **p < 0.01. Data in C represented the mean ± SD from 12 animals from two independent experiments (*N* = 12). Bars: 1.0 cm.

To better characterize the functionality of miR-552 in CRC tumorigenesis *in vitro* and *in vivo*, LOVO and LS174T cells expressing miR-552 inhibitor (antagomiR-552) were used for evaluating the impact of miR-552 on the capacities of clonogenicity *in vitro* (Figure 6) and xenograft tumorigenicity in BALB/C mice *in vivo* (Figure 7). Importantly, an inhibition of miR-552 expression in CRC cells by introducing miR-552 inhibitor exhibited a potential to significantly decrease the capacity of clonogenesis (Figure 6) and tumorigenesis (Figure 7) in both of LOVO and LS174T cells, as compared with those cells infected with LV-NC (*p <* 0.01). These results imply that miR-552 may play a pivotal role in governing the stemness of colorectal cancer stem cells, suggesting that miR-552 may be a novel target for preventing CRC.

### Knockdown of ADAM28 expression increases the cell proliferation, migration and clonogenicity in LOVO and LS174T CRC cells

**Figure 8:**
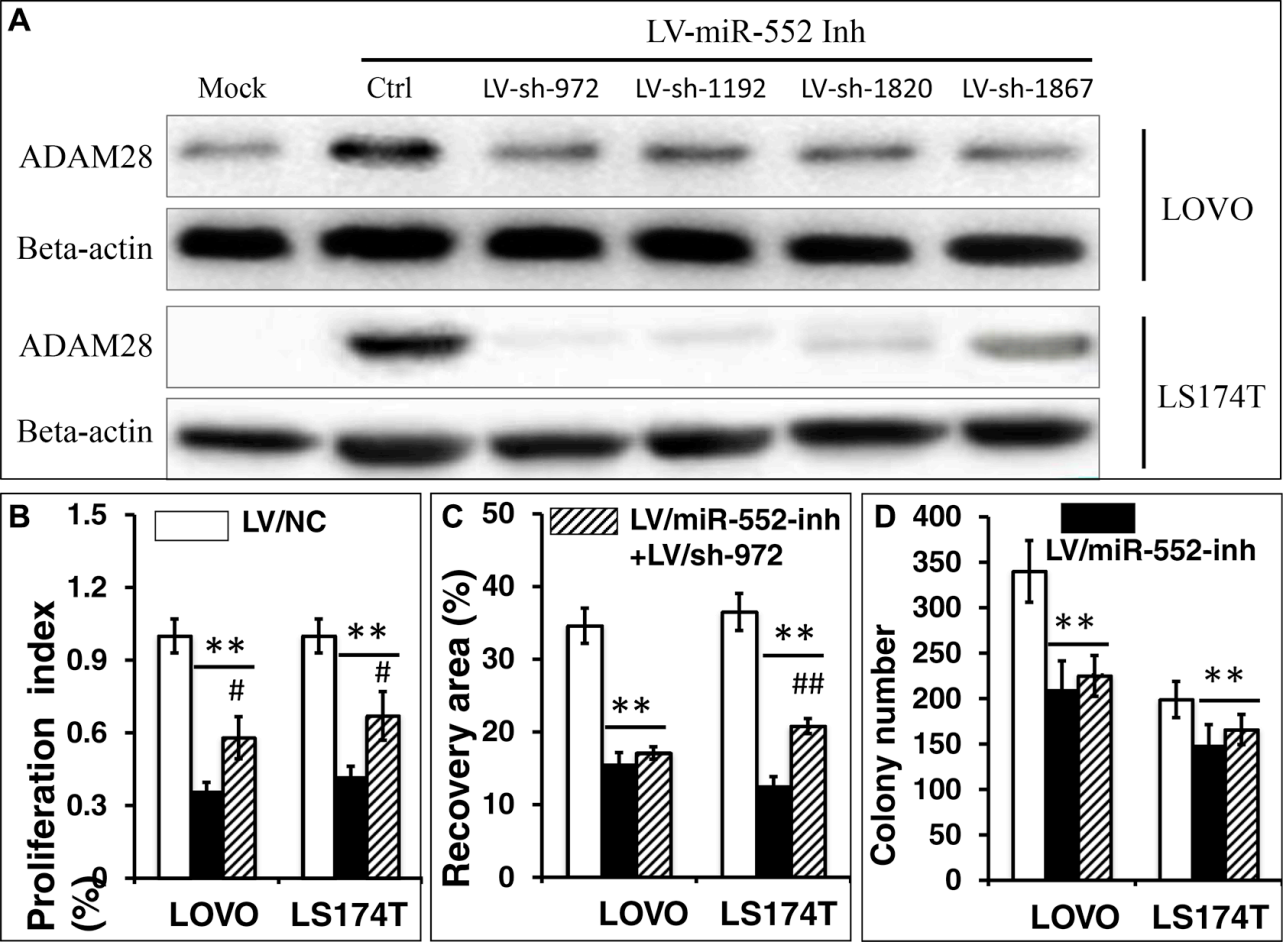
Knockdown of ADAM28 expression enhances the proliferation, migration and clonogenicity in LOVO and LS174T CRC cells LV-miR-552-inh transduced LOVO and LS174T cells were co-infected with one of LV-shADAM28-972, LV-shADAM28-1192, LV-shADAM28-1820 or LV-shADAM28-1867, the expression of ADAM28 protein was determined by an immunoblotting assay (**A**), and the capacity of proliferation (**B**), cell migration (**C**) and clonogenicity (**D**) were accessed in terms of an MTT assay, a scratch assay, and clone formation assay, respectively. (A) Representative blots of AMAM28 expression in LOVO cells (top two panels) and LS174T cells (bottom two panels) following shRNA interfaces. The immunoblotting assay showed the LV-shADAM28-972 could efficiently knockdown the LV-miR-552-inh-restored ADAM28 in both LOVO and LS174T cells, which was chosen for using in further analysis in this study. (B) An MTT assay showed a promotion of cell proliferation in LV-shADAM28-972 and LV-miR-552-inh-co-infected LOVO and LS174T cells (*p* < 0.05). (C) Relevant quantification of the results of cell migration index showed a significantly enhanced capacity of migration in LV-shADAM28-972 and LV-miR-552-inh-co-infected LS174T cells (*p* < 0.05), but not LOVO cells. (D) Relevant quantification of the results of cell clonogenic index, which showed a moderate increase of clonogenic capacity in LV-shADAM28-972 and LV-miR-552-inh-co-infected cells (*p* > 0.05). Compared with LV-NC group, *p < 0.01; **p < 0.01. Compared with LV-miR-552-inh, ^#^*p* < 0.01; ^##^*p* < 0.01. Data in B–D represented the mean ± SD from three independent triplicated experiments (*N* = 9).

In order to further examine whether the miR-552 promotes CRC cell metastasis by targeting ADAM28, the up-regulated ADAM28 in LV-miR-552-inh transduced LOVO and LS174T cells was knocked down by shRNAs to ADAM28 mRNA. To this end, LV-miR-552-inh transduced LOVO and LS174T cells were first infected with one of LV-shADAM28-972, LV-shADAM28-1192, LV-shADAM28-1820 or LV-shADAM28-1867, and the expression of ADAM28 protein was determined by an immunoblotting assay. The result showed that all the four tested shRNA vectors were able to reduced the expression of ADAM28 protein at various degrees in LV-miR-552-inh transduced LOVO and LS174T cells, among them the LV-shADAM28-972 displayed the most efficient knockdown of the targeted protein in both LOVO cells and LS174T cells (Figure 8A). The LOVO and LS174T CRC cells that co-infected with LV-shADAM28-972 vector and LV-miR-552-inh were employed for the study of loss-function of ADAM28, as an infection of LV-miR-552-inh resulted in an elevated ADAM28 expression in LOVO and LS174T cells (Figure 3C). Notably, the LV-shADAM28-972-mediated reduction of ADAM28 expression showed a significantly enhanced capacity of cell proliferation in LV-miR-552-inh-transduced LOVO and LS174T CRC cells (*p <* 0.05) (Figure 8B), and statistically increased ability of cell migration in LV-miR-552-inh-transduced LS174T cells (*p <* 0.05) but not in the LOVO cells (*p* > 0.05) (Figure 8C). However, only moderately enhanced ability clonogenicity in both LV-shADAM28-972 co-infected LV-miR-552-inh-transduced LOVO and LS174T CRC cells was observed (*p >* 0.05) (Figure 8D). These data indicated that a knockdown of ADAM28 expression could partially reverse the miR-552-inh-mediated inhibition of metastatic capacity of LOVO and LS174T CRC cells *in vitro,* suggesting that a oncogenic role of miR-552 in the CRC cells was in part through a mechanism by directly targeting *ADAM28* gene.

## DISCUSSION

An increased numbers of evidence has suggested that miRNAs play crucial roles in the development and progression of human CRC. In the present study, we identified miR-552 and miR-592 were potential oncogenes in CRC, in which both miR-552 and miR-592 were significantly evoked in CRC, which were correlated with the TNM stages and lymph node metastasis of this type of cancer. Importantly, enforced expression of miR-552 inhibitor, the antagomir-552 showed an ability to inhibit the proliferation, migration and clonogenicity of CRC cells *in vitro*, and to repress tumor growth in a xenograft tumor model of SCID mice *in vivo*. In contrast, a reduction of ADAM28 using shRNA was able to reverse the antagomir-552-mediated inhibition of metastatic properties of CRC cells. Mechanistically, the oncogenic role of miR-552 is at least in part through a mechanism by directly targeting *ADAM28* mRNA.

ADAM28 is an important member of the ADAM gene family, which shares a more homological sequence of amino acid with snake venom metalloproteinase (SVMPs) relative to other family members [16]. The metalloproteinase domain of ADAM28 has the zinc-binding consensus sequence, and ADAM28 exhibits catalytic activity to a few substrates such as IGFBP-3 and vWF [15]. A pronounced expression of ADAM28 was frequently observed in human solid tumors, such as lung cancer, breast cancer and bladder cancer [17–21]. For example, ADAM28 was overexpressed in human breast tumor tissues, which was positively correlated with the proliferative activity of the cancer cells. Furthermore, an exposure of breast carcinoma cells (MDA-MB231) to IGF-I led an enhanced capacity of cell proliferation, cleavage of IGFBP-3 and activation of IGF-I cell signaling. Importantly, these processes could be dramatically inhibited by a treatment of anti-ADAM28 antibody or small interfering RNA to ADAM28, suggesting that ADAM28 had a clinical implication in breast cancer cell proliferation through enhanced bioavailability of IGF-I released from the IGF-I/IGFBP-3 complex by selective IGFBP-3 cleavage [20]. With respect to lung carcinomas, the expression of ADAM28 was also elevated, suggesting that it may be a better serological and immunohistochemical marker for non-small-cell lung cancers (NSCLC) [19].

Indeed, Kuroda et al. found that the ADAM28 level in the NSCLC tissue and serum was 36.9-fold and 4.6-fold over that in the non-neoplastic lung tissue and serum, respectively. Intriguingly, such an evoked expression of ADAM28 was also found in the patients with recurrent NSCLC and/or lymph node metastasis, and was correlated with a poor disease-free survival [19]. In asbestos-related lung adenocarcinomas, however the expression of ADAM28 was even more elevated than non-asbestos related lung cancer adenocarcinoma [18]. Equally noteworthy, the level of ADAM28 in sera of advanced NSCLC patients was declined during a chemotherapeutic process, and was significantly correlated with the therapeutic responses and prognosis, indicating that the serum ADAM28 may be a reliable surrogate marker for predicting tumor response to a chemotherapeutic regimen and the survival in patients with advanced NSCLC [17]. Mechanistically, an effect ADAM28 on cancer cell metastasis was through by cleaving vWF as determined in terms of assays including the yeast two-hybrid analysis, knockdown of ADAM28 expression using shRNAs or small interface RNA (siRNA) to ADAM28, or inhibition of activity using neutralizing anti-ADAM28 antibody in a mouse tumor xenograft models [15]. In addition, oncoprotein v-src also showed an ability to induce ADAM28 expression in human carcinoma cell lines of the lung, breast, ovary, kidney and colon [37]. Conversely, in the present study, a down-regulation of ADAM28 by miR-552 or shRNA showed enhanced metastatic properties of CRC cells *in vivo,* and a stored expression of ADAM28 protein by miR-552-inh exhibited a potential to reduce metastatic capacity of CRC cells *in vitro* and *in vivo*. Of great interest, the knockdown of ADAM28 could only partially restored the metastatic ability inhibited by miR-552 inhibitor in CRC cells, suggesting that miR-552 might promote the metastasis by targeting ADAM28 and other signaling in CRC. In addition, the distinct biofunctions of ADAM28 observed in various cancer types may imply a cancer type context-dependent biological function of ADAM28 in CRC cells, which warrants further investigations.

In the CRC, up-regulation of ADAM28 and IGFBP-3 genes in was only detected in the normal tissue of overweight/obese patients with CRC as determined by a qRT-PCR assay, but the histopathologically normal surgical margin in specimens was not equal to the molecular margin [23]. However, a controversial result was reported by Abe et al., by whom a co-expression of ADAM28 and phosphorylated v-src was found in neoplastic cells of the breast, lung, and colon carcinomas and some adenomas of the colon, but not in nonneoplastic colon mucosa, as determined by an IHC analysis [37]. In the present study, lower levels of ADAM28 transcript and protein were observed in the CRC tumor tissues relative the matched adjacent non-tumor tissues as examined in terms of respective qRT-PCR assay and IHC staining analysis. This finding was in agreement with that observed by Nowakowska-Zajdel [23]. We reason that the discrepancy of ADAM28 expression in CRC tissues found in different groups may be in part attributed to different resources of anti-ADAM28 antibodies used in different studies.

Accumulating evidence has demonstrated a potential effectiveness for suppressing tumor growth and reversing drug resistance by targeting oncomirs using antisense miRNAs. In this regard, miR-23a and miR-21 have gained the most interest, owing to an aberrant expression of these miRNAs in CRC cells. For instance, inhibition of miR-21 by antisense miR-21 (antagomir-21) promoted the differentiation CRC HCT-116 or HT-29 cells, which was accompanied by significant decreases of the expression of colorectal cancer stem cell (CRSC) marker CD44, capacity of colonosphere formation, and Wnt signaling activity but an increase of PDCD4 expression [39]. Similarly, miR-23a was found to be elevated in 5-FU CRC cells, and its target APAF-1 along with caspases-3 and -7 were down-regulated in these cells. Interestingly, introduction of miR-23a antisense into 5-FU resistant cells showed an increased level of apoptotic protease activating facter-1 (APAF-1), along with an enhanced activation of caspase-3 and -7, subsequently enhanced the 5-FU induced apoptosis in these cells [40]. By harnessing a similar strategy, Ge et al. investigated the effect of antagomiR-27a in glioblastoma [41]. As aberrant miR-21 and miR-592 seen in CRC, more abundant miR-27a transcript was detected in specimens from glioblastoma comparing with normal human brain tissues. A transduction of antagomiR-27a (miR-27a inhibitor) showed an ability to significantly inhibit the invasion and proliferation of U87 glioblastoma cells, and reduce the growth of glioblastoma xenograft in SCID mice [41]. Consistent with these findings, our data also showed that an introduction of miR-552 inhibitor (antagomiR-552) led to a suppression of CRC cell proliferation and migration, and a reduced capacity of CRC cell clonogenicity and tumorigenicity *in vitro* and *in vivo*.

In summary, less abundant ADAM28 protein was detected in CRC tissues and cell lines, accompanied with an evoked miR-552 expression. The ADAM28 gene was identified as a direct target of miR-552. Remarkably, an inhibition of miR-552 by antagomiR-552 exhibited an ability to reduce metastatic potentials and capacity of clonogenicity *in vitro*, and decrease the ability of tumorigenicity of CRC cells *in vivo*. In contrast, a knockdown of ADAM28 in CRC cells by shRNA showed an enhanced capacity of cancer cell proliferation and migration *in vitro*. Results from this study suggest that miR-552 acts as an oncogene in CRC, which can promotes cancer metastasis through a mechanism in part by directly targeting ADAM28 in CRC. This study thus offers an insight in the mechanism underpinning the metastasis of CRC, which warranted for further investigation as a novel target for prognosis, prevention and treatment of this disease.

## MATERIALS AND METHODS

### Ethics statement

Human colorectal tissue was collected with a protocol approved by the Ethic Committee for the Conduct of Human Research at Ningxia Medical University. Written consent was obtained from every individual according to the Ethic Committee for the Conduct of Human Research protocol. All participants were over 18 years of age and provided written informed consent for the publication of the data. The Human Research Ethic Committee at Ningxia Medical University approved this study.

### Human CRC tissue samples and cell lines

Fifty tumor samples with histologic evidence of CRC and matched adjacent non-tumor tissues were archival samples from department of Medical Pathology Department, General Hospital of Ningxia Medical University from the year of 2014 (Table 2). The pathologic tumor staging was determined according to the International Union Against Cancer (2009). Cell lines of human embryonic kidney 293, human CRC cell lines HCT116, LOVO, LS174T, SW480 and normal intestinal epithelial cell line CCD-18Co were purchased from American Type Culture Collection (Mannasas, VA, USA). The cells were cultured and maintained at 37°C in a humidified atmosphere of 5% CO2 95% air in dulbecco's modified eagle medium (DMEM) supplemented with 10% Fetal Bovine Serum (FBS) and 1% pen/strep.

**Table 2:** Demographics of patients with CRC and expression of miR-552

Demographics	Number of subjects	*p* value
Genders		0.222
Male	34	
Female	16	
Ages		0.117
≤ 62	26	
> 62	24	
Degrees of differentiation		0.115
High	14	
Medium	22	
Low	14	
TNM stages		0.001**
I	5	
II	26	
III	15	
IV	4	
Lymphonode metastasis		0.000***
Yes	22	
No	28	

Data was presented as mean ± SD.

### Immunohistochemistry staining

The expression of ADAM28 in clinic human CRC and matched adjacent non-tumor tissues was evaluated by an immunohistochemistry (IHC) staining using rabbit anti-ADAM28 antibody (1: 200 from Abcam, USA). The archival paraffin-embedded sections (5 μm) were deparaffinized and rehydrated through graded alcohol solution. Tissue sections were microwaved in 10 mM sodium citrate pH 6.0 for 15 minutes and cooled down to room temperature (RT) for antigen retrieval. Followed by treating sections with 0.3% hydrogen peroxide in phosphate buffered saline (PBS) for 15 minutes to inactivate endogenous peroxidase before they were blocked with blocking buffer (5% sheep serum in PBS) for 2 h at RT. The primary antibody was then applied (1:200 in blocking buffer) on the section and incubated overnight at 4°C. Paralleled sections incubated with normal rabbit IgG was used for negative controls. After washing for 3 × 5 min in PBS, sections were incubated with peroxidase labeled donkey anti-rabbit IgG (ZSGB-Bio Origene, Beijing, China) (1:500 in blocking buffer) for 30 minutes at RT. The ADAM28 signal was developed with 3, 3'-diaminobenzidine (DAB) peroxidase substrate, followed by counterstaining with hematoxylin if it was applicable. The stained sections were examined and photographed on a Nikon Optiphot II microscope equipped with a camera. The non-counterstained sections were also randomly imaged using a 10× objective lens for five fields of each section, and five sections for each sample were evaluated. The obtained images were then for a semi-quantitative analysis of the ADAM28 expression by measuring the integrated absorbance (IA) using image analysis software Image-Pro Plus 6.0 (IPP6.0, Media Cybernetics, Silver Spring, MD, USA), and the average of the IA values of each sample was used as an index of the expression of ADAM28 expression [42].

### Experimental validation of miR-552 target

In order to validate the ADAM28 mRNA is a target of miR-552 (MIMAT0003215), a reporter plasmid containing luciferase with the 3′UTR sequence of human ADM28 mRNA (GenBank database NM_014265.4), a fragment harboring gi|98985827:2412-3220 homo sapiens ADAM metallopeptidase domain 28 (ADAM28), transcript variant 1, mRNA was amplified, which included a miR-552 targeting sequence ACCTGT at position of ADAM28 3′-UTR. By employing a RT-PCR method, a wild-type and mutated 3′UTR of ADAM28 mRNA were amplified from RNA isolated from LOVO cells. The wild-type and mutated 3′UTR fragment were then cloned into the downstream of luciferase reporter gene of pMIR-Report vector (Invitrogen, Grand island, NY, USA). The respective ADAM28 mRNA luciferase reporter vectors, pMIR-Report/ADAM28 (harboring wild-type 3′UTR) and pMIR-Report/Mut-ADAM28 (containing a mutated 3′UTR) were then generated. The specificity of miR-552 targeting ADAM28 mRNA was ascertained by co-transfection plasmid DNA of pSicoR/miR-552, miR-552/inhibitor or pSicoR/NC and pMIR-Report/ADAM28 or pMIR-Report/Mut-ADAM28 into 293T cells and determined in terms of the relative activity of firefly luciferase unit (RLU) at 48 h post-transfection using a dual-luciferase Reporter assay kit (Promega, Madison, WI, USA). A Renilla luciferase expressing plasmid pRL-TK (Promega, Madison, WI, USA) was always included in the transfection to normalize the efficiency of each transfection.

### Quantitative reverse transcription-PCR (qRT-PCR)

Total RNA from infected cells was isolated with a Miniprep kit, and small RNA was purified with an RNAiso kit per manufacturer's recommendations (Takara, Dalian, China). Total RNA from archival paraffin sections was isolated using a Mag-Bind^®^ FFPE RNA Kit (Omega Bio-Tek Norcross, GA, USA). The purified RNA was then used for reverse transcription of the first-strand cDNA synthesis by reverse transcription using M-MLV reverse transcriptase. The expression levels of miRNAs were assessed by the stem-loop RT-PCR method using the Hairpin-it^™^ miRNAs. The sequence of the primer used for reverse transcription of mature miR-552 included a stem-loop structure, which was designed based on the sequence of miR-552 (Table 3). The sequence of primer set for amplification of miR-552 and U6 promoter were listed in Table 3. The U6 promoter was included and used to normalize for sample loading and RNA abundance. The qRT-PCR was performed using a Bio-Rad iQ5 lightcycler using a TaKaRa SYBR RT-PCR kit (Takara, Dalian, China). Relative expression was calculated as previously described using real-time PCR efficiencies and the crossing point deviation of unknown sample *vs* control using the ΔΔ Ct method [43]. The specificity of the primer sets was determined by sequencing the product of each qRT-PCR reaction.

**Table 3:** The sequences of primers used for reverse transcription and PCR

Application	Primer	Sequence (5′→3′)
Reverse transcription	miR-552 RT	GTCGTATCCAGTGCAGGGTCCGAGGTATTCGCAC TGGATACGACTTGTCT
	U6 RT	AACGCTTCACGAATTTGCGT
qRT-PCR of miR-552	Forward	CCGCACAGGTGACTGGTTAGA
	Reverse	GTGCAGGGTCCGAGGT
qRT-PCR of U6	U6 promoter forward	CTCGCTTCGGCAGCACA
	U6 promoter reverse	AACGCTTCACGAATTTGCGT

### Generation and infection of lentiviral vectors expressing miR-552 inhibitor and shRNAs to ADAM28

In order to suppress endogenous miR-552 in CRC cells, lentiviral vector expressing miR-552 inhibitor or scramble mRNA were generated by Shanghai GeneMarker Inc (Shanghai, China). This viral vector was designated as LV-miR-552-inh and was generated according to the sequence of hsa-miR-552-3p MIMAT0003215 (AACAGGUGACUGGUUAGACAA). The core sequence of LV-miR-552-inh was 5′-TTGTCTAACCAGTCACC TGTT). Meanwhile, a control Lentiviral vector LV-NC (core sequence: 5′-TTCTCCGAACGTGTCACGTTTC-3′) was also generated. For construction of the lentiviral vector expressing miR-552-inh, oligonucleotides of sense strand (5′-GATCCGTTGTCTAACCAGTCACCTGTTTCAAGA GAACAGGTGAGGTTAGACAACTTTTTTG-3) and antisense strand (5′-AATTCAAAAAAGTTGTCTAACC TCACCTGTTCTCTTGAAACAGGTGACTGGTTAGA CAACG-3′) were synthesized, which were based on the sequence of human miR-552 from miRBase database. Restriction endonuclease *BamH* I and *EcoR* I were introduced at 5′-ends of these oligonucleotides, respectively. The mixture of the sense and anti-sense oligonucleotides was then used for production of the precursor of short hairpin RNA (shRNA) of miR-552 inhibitor by temperature annealing approach. The miR-552 inhibitor precursor was modified with appropriate restriction enzymes, and cloned into a miRNA expressing plasmid, pSicoR (Department of Biological Chemistry, School of Medicine, Fudan University, Shanghai, China) to generate the vector expressing miR-552 inhibitor, which was designated as pSicoR/miR-552-inh in this study. Using the same approach, a negative control vector (pSicoR/NC) was also generated using oligonucleotides of 5′-GATCCGTTCTCCGAACGTGTCACGTTTCAA GAGAACGTGACACGTTCGGAGAACTTTTTTG-3′ for sense strand and 5′-AATTCAAAAAAGTTCTCCGAAC GTGTCACGTTCTCTTGAAACGTGACACGTTCGGA GAACG-3′ for antisense strand. In order to knockdown the expression of ADAM28, four lentiviral vectors expressing shRNAs to human ADAM28 were generated based on the coding sequence (cds) of ADAM28 provided in GenBank (BC136478.1). These shRNAs were designed to respectively target the coding sequence of 5′-GGAC CACAGCGATAATCTTCT-3′, 5′-GCTCCATTGCCTAC AGATATC-3′, 5′-GCGATAACAAGGTTTGCATTA-3′ and 5′-GCCTACAAATCAACCAATTGC-3′ of ADAM28 mRNA. These shRNA viral vectors were designated as LV-shADAM28-972 (the 5′-end nucleotide position from the start codon of ADAM28 mRNA), LV-shADAM28-1192, LV-shADAM28-1820 and LV-shADAM28-1867, respectively. For production of the lentiviral vectors, 1 × 10^6^ HEK 293T cells were seeded per well in six-well plates with 2 mL of DMEM/10% FBS without antibiotics. The next day, the medium was replaced with 1 mL DMEM without FBS and antibiotics. Subsequently, the proviral vector (1.5 μg) was co-transfected with packaging plasmids pCMV-VSV-G (0.5 μg) and pCMV-dR8.91 (1 μg) (Department of Biological Chemistry, School of Medicine, Fudan University, Shanghai, China) with TransLipid Transfection Reagent as suggested by the manufacturer. The medium was replaced with 2 mL of DMEM/10% FBS at 6 h post transfection. The supernatant was harvested at 48 h after transfection, followed by being filtered through a 0.45-μm pore size filter, and then concentrated to 1/100 volume by ultracentrifugation with 50000 × g at 4°C for 2.5 h using a SW28 rotor (Beck-man Coulter, Fullerton, CA, USA) and Sorvall Ultra 80^®^ (Kendro Laboratory Products, Newtown, CT, USA). The virus particle pellet was resuspended in PBS and frozen at −80°C till use. The viral particles were titrated in 293T cells by counting EGFP-positive cells, CRC LOVO and LS174 cells were respectively infected with each of above viral vectors, and continuously cultured for 3 days before being used, or screened in media containing 2.5 μg/mL Puromycin for additional one week for removing un-transduced cells, the cell pools were then collected and employed for further studies.

### Immunoblotting analysis

Whole cell lysates (75 μg) or homogenized tissues were prepared in a lysis buffer (50 mM Tris-HCl, pH 7.5, 5 mM EDTA, 150 mM NaCl, 0.5% NP-40). Protein samples were resolved on a 10% sodium dodecyl sulfate (SDS)-polyacrylamide gel (SDS-PAGE), followed by being transferred to a PVDF membrane (Millipore, USA). The membranes were then probed with mouse anti-ADAM28 antibody (Abcam, USA) and anti-beta-actin antibody (Boster, Wuhan, China) were for the interested protein ADAM28 and endogenous beta-actin for loading control, respectively. The blots were developed using the enhanced chemiluminescence (ECL) reagent (Advansta, Menlo Park, CA, United States) after they were incubated with the appropriate peroxidase labeled secondary antibodies. The levels of protein expression were semi-quantified by optical densitometry using ImageJ Software version 1.46 (http://rsb.info.nih.gov/ij/). The ratio between the net intensity of each sample divided by the β-actin internal control was calculated as densitometric arbitrary units (A.U.)) which served as an index of relative expression of a protein of interest [42, 44].

### MTT assay

Cell proliferation was determined by using the MTT cell proliferation kit (Solarbio, Beijing, China). Lentiviral infected LOVO or LS174T cells were split and seeded in each 96-well plate at a density of 2 × 10^4^ per well and allowed to adhere overnight. The cells were then used for MTT assay at indicated time points per the manufacturer's instruction (Bio-Rad Laboratories, Inc., Irvine, CA, USA).

### Cell migration assay

The lentiviral infected CRC cells were seeded at 80% confluent and infected with lentiviral vectors for 48 h (cells were grown to confluence) in 6-well culture plates. The cells were then scratched with 200 ml pipette tips. The resultant unattached cells were removed by washing with pre-warmed PBS for three times, the wounded monolayers were cultured for additional 48 h prior to be stained with 0.1% crystal violet solution. The closure of the wounded areas was observed under a microscope at magnification of 40× (Leica, Germany) and photographed. The distance of closure was quantified with the NIH Image J image processing program. These experiments were performed in triplicate. Each condition was tested in duplicate and each experiment was repeated at least three times.

### Clonogenic assay

A clonogenic assay was used for accessing the stemness of CRC cells. For analysis of clonogenicity, single cell suspension of 700 cells were seed on 35-mm dishes. Cells were continuously cultured for 10 days with refreshment of medium 3 days interval. For colony counting, the medium was removed and the cells were rinsed with PBS prior to be fixed with 4% paraformaldehyde at room temperature for 5 min. After removing the fixation solution, the cells were then stained with 0.5 % crystal violet solution and incubate at room temperature for 30 min. The staining solution was carefully removed, and the cells were rinsed with H2O to remove residual staining solution before air-dry the plate at room temperature for up to a day. Count number of colonies and calculate under a light microscope. Each condition was tested in duplicate and each experiment was repeated for three times.

### *In vivo* analysis of tumorigenic capacity of CRC cells

The tumorigenic capacity of CRC cells was evaluated in 6–8 week-old of female SCID mice. The mice were subcutaneously inoculated (s.c.) with 200 μL Matrigel (BD) containing 1 × 10^6^ of CRC cells (LV-NC or LV-miR-552-inh transduced). The animals were euthanized and the tomor tissues of injection sites were collected for evaluation of tumorigenicity after 3 weeks following the cell transplantation. The diameter of tumor was measured and used as an index of the tumorigenicity.

### Statistical analysis

All data collected in this study was obtained from at least three independent experiments for each condition. SPSS17.0 analysis software (SPSS Inc., Chicago, IL, USA) and PRISM 5 (GraphPad software, La Jolla, CA, USA) were used for the statistic analysis. Statistical evaluation of the data was performed by one-way ANOVA when more than two groups were compared with a single control, and *t*-test for comparison of differences between the two groups. Significant differences were assigned to *p* values < 0.05, < 0.01 and < 0.0001 denoted by *, ** and ***, respectively. Data was presented as the mean ± standard deviation (SD).

## SUPPLEMENTARY MATERIALS





## References

[1] Siegel RL, Miller KD, Jemal A (2015). Cancer statistics, 2015. CA Cancer J Clin.

[2] Garofalo M, Croce CM (2013). MicroRNAs as therapeutic targets in chemoresistance. Drug resistance updates.

[3] Cojoc M, Mabert K, Muders MH, Dubrovska A (2015). A role for cancer stem cells in therapy resistance: cellular and molecular mechanisms. Seminars in cancer biology.

[4] Kartal-Yandim M, Adan-Gokbulut A, Baran Y (2015). Molecular mechanisms of drug resistance and its reversal in cancer. Critical reviews in biotechnology.

[5] Lippert TH, Ruoff HJ, Volm M (2008). Intrinsic and acquired drug resistance in malignant tumors. The main reason for therapeutic failure. Arzneimittel-Forschung.

[6] Wang J, Du Y, Liu X, Cho WC, Yang Y (2015). MicroRNAs as Regulator of Signaling Networks in Metastatic Colon Cancer. BioMed research international.

[7] Bao Y, Chen Z, Guo Y, Feng Y, Li Z, Han W, Wang J, Zhao W, Jiao Y, Li K, Wang Q, Wang J, Zhang H (2014). Tumor suppressor microRNA-27a in colorectal carcinogenesis and progression by targeting SGPP1 and Smad2. PloS one.

[8] Di Leva G, Garofalo M, Croce CM (2014). MicroRNAs in cancer. Annual review of pathology.

[9] Christian LM (2012). The ADAM family: Insights into Notch proteolysis. Fly.

[10] Kelwick R, Desanlis I, Wheeler GN, Edwards DR (2015). The ADAMTS (A Disintegrin and Metalloproteinase with Thrombospondin motifs) family. Genome biology.

[11] Zhang P, Shen M, Fernandez-Patron C, Kassiri Z (2015). ADAMs family and relatives in cardiovascular physiology and pathology. Journal of molecular and cellular cardiology.

[12] Cal S, Lopez-Otin C (2015). ADAMTS proteases and cancer. Matrix biology.

[13] Mochizuki S, Okada Y (2007). ADAMs in cancer cell proliferation and progression. Cancer science.

[14] Sun Y, Huang J, Yang Z (2015). The roles of ADAMTS in angiogenesis and cancer. Tumour biology.

[15] Mochizuki S, Soejima K, Shimoda M, Abe H, Sasaki A, Okano HJ, Okano H, Okada Y (2012). Effect of ADAM28 on carcinoma cell metastasis by cleavage of von Willebrand factor. Journal of the National Cancer Institute.

[16] Mochizuki S, Okada Y (2009). ADAM28 as a target for human cancers. Current pharmaceutical design.

[17] Lv YL, Yuan DM, Wang QB, Zhan P, Luo L, Lv TF, Liu HB, Li YF, Wen J, Song Y (2012). Baseline and decline of serum ADAM28 during chemotherapy of advanced non-small cell lung cancer: a probable predictive and prognostic factor. Medical oncology.

[18] Wright CM, Larsen JE, Hayward NK, Martins MU, Tan ME, Davidson MR, Savarimuthu SM, McLachlan RE, Passmore LH, Windsor MN, Clarke BE, Duhig EE, Yang IA (2010). ADAM28: a potential oncogene involved in asbestos-related lung adenocarcinomas. Genes, chromosomes & cancer.

[19] Kuroda H, Mochizuki S, Shimoda M, Chijiiwa M, Kamiya K, Izumi Y, Watanabe M, Horinouchi H, Kawamura M, Kobayashi K, Okada Y (2010). ADAM28 is a serological and histochemical marker for non-small-cell lung cancers. International journal of cancer Journal international du cancer.

[20] Mitsui Y, Mochizuki S, Kodama T, Shimoda M, Ohtsuka T, Shiomi T, Chijiiwa M, Ikeda T, Kitajima M, Okada Y (2006). ADAM28 is overexpressed in human breast carcinomas: implications for carcinoma cell proliferation through cleavage of insulin-like growth factor binding protein-3. Cancer research.

[21] Tyan YC, Yang MH, Chen SC, Jong SB, Chen WC, Yang YH, Chung TW, Liao PC (2011). Urinary protein profiling by liquid chromatography/tandem mass spectrometry: ADAM28 is overexpressed in bladder transitional cell carcinoma. Rapid communications in mass spectrometry.

[22] Zhang XH, Wang CC, Jiang Q, Yang SM, Jiang H, Lu J, Wang QM, Feng FE, Zhu XL, Zhao T, Huang XJ (2015). ADAM28 overexpression regulated via the PI3K/Akt pathway is associated with relapse in de novo adult B-cell acute lymphoblastic leukemia. Leukemia research.

[23] Nowakowska-Zajdel E, Mazurek U, Wierzgon J, Kokot T, Fatyga E, Ziolko E, Klakla K, Blazelonis A, Waniczek D, Glogowski L, Kozowicz A, Niedworok E, Muc-Wierzgon M (2013). Expression of ADAM28 and IGFBP-3 genes in patients with colorectal cancer - a preliminary report. International journal of immunopathology and pharmacology.

[24] Mochizuki S, Tanaka R, Shimoda M, Onuma J, Fujii Y, Jinno H, Okada Y (2010). Connective tissue growth factor is a substrate of ADAM28. Biochemical and biophysical research communications.

[25] Shimoda M, Hashimoto G, Mochizuki S, Ikeda E, Nagai N, Ishida S, Okada Y (2007). Binding of ADAM28 to P-selectin glycoprotein ligand-1 enhances P-selectin-mediated leukocyte adhesion to endothelial cells. The Journal of biological chemistry.

[26] Miyamae Y, Mochizuki S, Shimoda M, Ohara K, Abe H, Yamashita S, Kazuno S, Ohtsuka T, Ochiai H, Kitagawa Y, Okada Y (2016). ADAM28 is expressed by epithelial cells in human normal tissues and protects from C1q-induced cell death. The FEBS journal.

[27] Liu Y, Wu C, Wang Y, Wen S, Wang J, Chen Z, He Q, Feng D (2014). MicroRNA-145 inhibits cell proliferation by directly targeting ADAM17 in hepatocellular carcinoma. Oncology reports.

[28] Wan D, Shen S, Fu S, Shen C, Wu J, Wang S, Xie W, Chen B, A L, Guo Y, Zheng D, Zhi Q, Peng B (2015). miR-203 suppresses the proliferation and metastasis of hepatocellular carcinoma by targeting oncogene ADAM9 and oncogenic long non-coding RNA HULC. Anti-cancer agents in medicinal chemistry.

[29] Zhang J, Qin X, Sun Q, Guo H, Wu X, Xie F, Xu Q, Yan M, Liu J, Han Z, Chen W (2015). Transcriptional control of PAX4-regulated miR-144/451 modulates metastasis by suppressing ADAMs expression. Oncogene.

[30] Kim J, Lim NJ, Jang SG, Kim HK, Lee GK (2014). miR-592 and miR-552 can distinguish between primary lung adenocarcinoma and colorectal cancer metastases in the lung. Anticancer research.

[31] Xia ZS, Wang L, Yu T, Zhong W, Lian GD, Wu D, Zhou HM, Chen GC (2014). MiR-5000-3p, miR-5009-3P and miR-552: potential microRNA biomarkers of side population cells in colon cancer. Oncology reports.

[32] Wang XY, Wu MH, Liu F, Li Y, Li N, Li GY, Shen SR (2010). Differential miRNA expression and their target genes between NGX6-positive and negative colon cancer cells. Molecular and cellular biochemistry.

[33] Fu Q, Du Y, Yang C, Zhang D, Zhang N, Liu X, Cho WC, Yang Y (2016). An oncogenic role of miR-592 in tumorigenesis of human colorectal cancer by targeting Forkhead Box O3A (FoxO3A). Expert opinion on therapeutic targets.

[34] Chen WC, Lin MS, Ye YL, Gao HJ, Song ZY, Shen XY (2012). microRNA expression pattern and its alteration following celecoxib intervention in human colorectal cancer. Experimental and therapeutic medicine.

[35] Liu M, Zhi Q, Wang W, Zhang Q, Fang T, Ma Q (2015). Up-regulation of miR-592 correlates with tumor progression and poor prognosis in patients with colorectal cancer. Biomedicine & pharmacotherapy = Biomedecine & pharmacotherapie.

[36] Liu Z, Wu R, Li G, Sun P, Xu Q, Liu Z (2015). MiR-592 inhibited cell proliferation of human colorectal cancer cells by suppressing of CCND3 expression. International journal of clinical and experimental medicine.

[37] Abe H, Mochizuki S, Ohara K, Ueno M, Ochiai H, Kitagawa Y, Hino O, Sato H, Okada Y (2013). Src plays a key role in ADAM28 expression in v-src-transformed epithelial cells and human carcinoma cells. The American journal of pathology.

[38] Jowett JB, Okada Y, Leedman PJ, Curran JE, Johnson MP, Moses EK, Goring HH, Mochizuki S, Blangero J, Stone L, Allen H, Mitchell C, Matthews VB (2012). ADAM28 is elevated in humans with the metabolic syndrome and is a novel sheddase of human tumour necrosis factor-alpha. Immunology and cell biology.

[39] Yu Y, Sarkar FH, Majumdar AP (2013). Down-regulation of miR-21 Induces Differentiation of Chemoresistant Colon Cancer Cells and Enhances Susceptibility to Therapeutic Regimens. Translational oncology.

[40] Shang J, Yang F, Wang Y, Wang Y, Xue G, Mei Q, Wang F, Sun S (2014). MicroRNA-23a antisense enhances 5-fluorouracil chemosensitivity through APAF-1/caspase-9 apoptotic pathway in colorectal cancer cells. Journal of cellular biochemistry.

[41] Ge YF, Sun J, Jin CJ, Cao BQ, Jiang ZF, Shao JF (2013). AntagomiR-27a targets FOXO3a in glioblastoma and suppresses U87 cell growth *in vitro* and *in vivo*. Asian Pacific journal of cancer prevention.

[42] Li H, Li C, Dai R, Shi X, Xu J, Zhang J, Zhou X, Li Z, Luo X (2012). Expression of acetylated histone 3 in the spinal cord and the effect of morphine on inflammatory pain in rats. Neural regeneration research.

[43] Pfaffl MW (2001). A new mathematical model for relative quantification in real-time RT-PCR. Nucleic Acids Res.

[44] Li T, Yang J, Lv X, Liu K, Gao C, Xing Y, Xi T (2014). miR-155 regulates the proliferation and cell cycle of colorectal carcinoma cells by targeting E2F2. Biotechnology letters.

