# Supplementary material for: MicroRNA-552 enhances metastatic capacity of colorectal cancer cells by targeting a disintegrin and metalloprotease 28
Source: Oncotarget. 2016 Sep 21;7(43):70194–210. doi: 10.18632/oncotarget.12169 (PMC5342546; doi:10.18632/oncotarget.12169)
Supplement: Supplementary file 1 [file oncotarget-07-70194-s001.pdf]

MicroRNA-552 enhances metastatic capacity of colorectal cancer cells by targeting a disintegrin and metalloprotease 28

Supplementary Materials

Supplementary Table S1: Potential targets of miR-552 predicted TargetScan online software.
See Supplementary_Table_S1
